# Supplementary figures and images for: Retinal optic flow during natural locomotion
Source: PLoS Comput Biol. 2022 Feb 22;18(2):e1009575. doi: 10.1371/journal.pcbi.1009575 (PMC8896712; doi:10.1371/journal.pcbi.1009575)

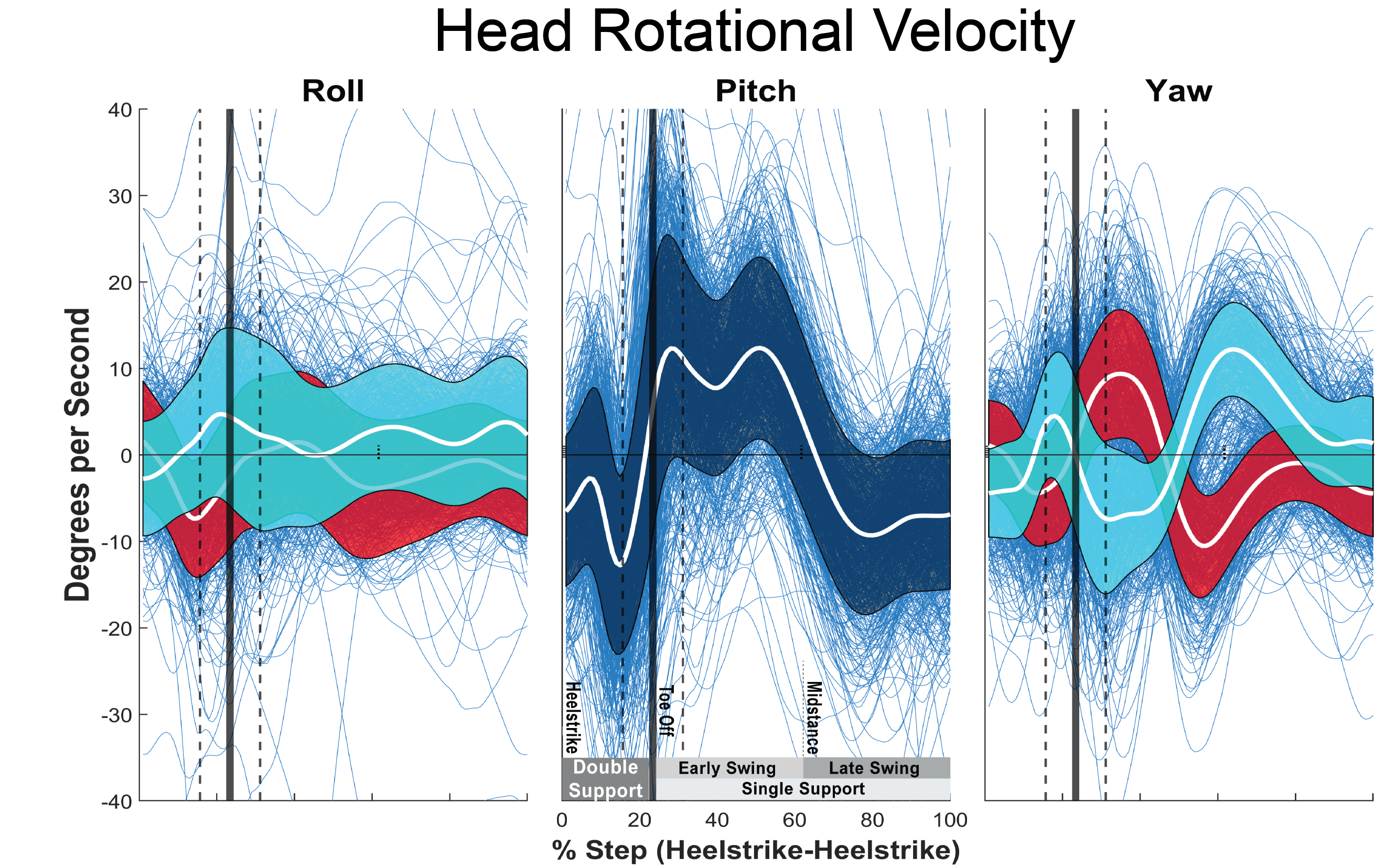

Supplement: S1 Fig — Head rotational velocity throughout the course of the gait cycle. Measured similarly to the the acceleration values reported in Fig 4, but using rotational velocity measured by the triaxial gyroscope in the head-mounted IMU. Note that gyroscopes are generally noisier and less reliable than linear accelerometers. (TIF) [file pcbi.1009575.s015.tif]
