# Supplementary material for: Retinal optic flow during natural locomotion
Source: PLoS Comput Biol. 2022 Feb 22;18(2):e1009575. doi: 10.1371/journal.pcbi.1009575 (PMC8896712; doi:10.1371/journal.pcbi.1009575)
Supplement: Attachment — Submitted filename: RetinalFlow_Comments to the Authors_Final.pdf [file pcbi.1009575.s016.pdf]

We would like to thank the reviewers for their careful reading and thoughtful suggestions. The manuscript has been substantially altered based on the recommendations, including several new figures and two new appendices. Changes to the text is indicated by blue font in the attached manuscript. Our replies to specific comments are included in blue font below

Comments to the Authors:

Reviewer #1: This paper reports the results of a study in which participants' gaze were monitored while walking over terrain with a head mounted camera viewing the scene, allowing for the first ever recording of time-varying images on the retina during natural locomotion. The authors analyze the optic flow patterns, revealing interesting and highly non-trivial patterns that could be used as cues in guiding navigation.

This is a landmark study, sure to cause many people in the field to rethink what optic flow means, and how it is used during natural vision. A rich set of findings, beautifully analyzed, and the videos are fantastic. This is sure to get a lot of attention and motivate follow up studies, etc. It is a paper that really forces you to think as the results are not obvious.

Some general comments:

You describe multiple conditions for the locomotion, mulch vs. rocky terrain, how the subject is looking ahead, etc. But I don't see that the results of these different conditions are separated out, they all seem lumped together somehow. There is a figure inset in Figure 2, but I didn't see anything else. Is it because there are no appreciable differences?

The conditions in this study were mostly selected for the behavior they evoked in anticipation of later analysis (e.g. 'Distant Fixation' was designed to provide the most 'psychophysics-like' behavior). Indeed, there were not many notable differences in the behaviors relevant to this manuscript (and detailed analysis of these differences is beyond the scope of this paper). To clarify this point, additional discussion was added around Lines 102-116.

In addition, we pulled the inset figure from Figure 2 into its own Figure 3, and added a brief discussion at Lines 144-148 explaining that FoE velocity was not notable different between conditions (likely due to the fact that the underlying movement patterns of locomotion weren't significantly altered by the behaviors in the different conditions)

Figure 2 shows a histogram of the rapid speeds at which the FOE moves in head centered reference frame. 1000 deg/sec seems remarkably high, perhaps worth commenting on this.

It certainly is! Although a full geometric/optical analysis of the way these velocities arise during natural locomotion is unfortunately beyond the present scope of work, we added some more discussion of the intuitive reason for these high velocities throughout Section 2.1.1-2.1.2.

In short, the high FoE velocity seen here arises from the fact that the head-centered FoE at a given moment will arise from the direction of the head's translation vector at that moment. FoE velocity arises from the angular velocity of the head's translation vector as it changes over time. Historically, vision scientists have used experimental displays that implicitly assume that locomotion is well approximated by a constant velocity straight-line motion (occasionally with smooth curvilinear movement or added 'jitter' to represent locomotion). This assumption led researchers to underestimate the magnitude of change in the head's velocity vector throughout the gait cycle, and led to the belief that head-centered velocity is more stable than retinal velocity. In the present manuscript, we show that the head's translation vector changes dramatically throughout the gait cycle (Figure 4, and the yellow vector in Video3), which will accordingly result in large changes in the head-centered FoE.

Also it would be nice to see the corresponding histogram in the retinal case - it should be much sharper and peaked near zero right?

We chose not to include this due to the fact that our method for 'idealizing' fixation would cause this number to artificially peak at zero, and calculation of this number based on the empirically measured gaze position would incorporate eye tracker noise (and it is currently unknown how much of the fixational drift is the result of physiology vs inaccuracy inherent to the current iteration of eye tracking technology). Instead, we have added an Appendix which discusses the reasoning behind (and

consequences of) our gaze idealization method, which includes a report of the actual empirically measured fixational drift values (see reply to your next point)

The manner in which images are cast into retinal coordinates seems a bit strange - i.e., assuming "perfect" fixation etc. I think you should define for the reader what perfect fixation means. I'm not sure just from reading everything in the methods that I could exactly replicate what the authors did, I think it could use spelling out more.

Yes, we agree that this is important and deserves a more detailed exposition, which we have now done both in the main text and in Appendix A. We had previously calculated how well gaze was stabilized but mentioned it only in a footnote. We now describe this more extensively and present the data in the Appendix. In short - We used the DeepFlow algorithm on the video record to measure residual image motion near the fovea during a fixation. We showed that the mode of retinal image slip during a fixation is around 0.25 deg of visual angle, with a median slip of less than 0.82 deg. We also show that the direction of the slip is mostly downward, as expected from imperfect stabilization (and/or parallax error from the eye tracker, see Evans et al 2012). Our thought was that this was very good, especially given the expected instability even during head-fixed fixation, eye tracker noise, and the inexact nature of foot placement. (A foothold subtends about 2-4 deg laterally at approximately 2 steps ahead.)

In addition to this, we now calculate how 4 deg per sec of added motion down and to the left (1 deg of slip during a 250 msec fixation) would change the div and curl estimates (Appendix A Fig 2). It is hard to evaluate how significant that would be without knowing exactly how the curl/div signal is used in practice. (For example, use of the peak div requires knowledge of gaze angle relative to the body, which has some associated noise.) At this point we cannot make a strong case about how the calculated regularities are used and have made this clearer in the manuscript (see also response to Reviewer 2 below). Irregularities in the ground plane will distort the flow patterns as well as fixational instabilities, so we are simply measuring the structure of the retinal motion patterns in the best-case scenario, and suggesting that it might be used. Showing that/how it is used is beyond the scope of the current paper.

Section 2.1.3: "When gaze is properly stabilized on a location in the world during locomotion, the result will always be a pattern of radial expansion combined with rotation, centered on the point of fixation," - but in the intro I think you say this: "The resulting flow field will comprise both a rotational and a translational component," –

We agree that this wording seems a bit inconsistent as written. We altered the wording at both locations to clarify our point.

curl and divergence: I think it would be worth providing a short recap for those of us rusty on our calculus about what these things measure exactly, and how they are computed. Also I had thought curl produces a vector field, but in Figure 5 you show just a scalar field. And then there is the idea of "net curl" because the +'s cancel the -'s. Yet the divergence measure is just calculated locally and we don't have a "net div." Not sure how to think about all this, again a bit more mathematical explanation would be helpful here.

Per your suggestion, we added Appendix B that describes our calculation of Curl and Divergence in greater detail and provide an intuitive introduction to these topics (along with a new figure and sample Matlab/Octave commands to introduce readers to these topics).

Concerning your question about Curl being a vector – It is true that in the case of a 3D vector field, curl defines a vector denoting the 3D axis of rotation. However, when taking the curl of a 2d vector field (as we do here), rotation is assumed by convention to occur relative to an axis orthogonal to the 2d vector field. In the 2d case the curl operator produces a scalar value with sign denoting the direction of rotation and magnitude denoting rotational velocity (we also explain this point in the new Appendix).

Section 2.2.3: "The point of maximum divergence encodes the body's over-ground momentum vector in retinotopic coordinates" - if this is true, which I suppose it is, then shouldn't you be able to show this from your data? i.e., you have the body speed and you know where they are looking, so you should be able to make a joint scatter plot of these things showing a linear relationship, no? I think it would be quite compelling and help to bolster the point.

Similarly for the curl patterns - you argue that this could be a cue, but the evidence shown is rather anecdotal, we just see a few select plots. How about just show a joint scatter plot of curl vs. where the subject is fixating?

This is a great suggestion! We computed this analysis and included it in a new figure (Fig 7). As expected, Curl and Div show a linear relationship to the angle between the eye's velocity vector and the point of fixation. We agree that this simple visualization strengthens the point being made here.

Typos:

nulls image at the fovea, --> nulls image motion at the fovea, Done

However, not matter --> However, no matter Done

the the point --> the point

line:
14 - this claim may be true but I think it needs a citation. We softened the language to make it clear that this statement was based on our interpretation.

47 - 'judgements of their direction' Done
51 - 'no matter' Done
72 - 'apostrophy+t' misplaced - This is actually part of the surname of “Bernard ‘t Hart,” a vision scientist whose name is the bane of citation managers everywhere!

107 - period and semicolon together Done
185 - the left apostrophe is the right-side type
Figure 4 - should add labels of gamma, rho coordinates to figures, especially part a to demonstrate – Done.
276 - space before 'Because' - Done
299, 313, ,377, 378- maybe bold or italic instead of ** - Done
364 - space before 'Some' - Done
523 - 'Estimate' - Done
548 - remove space before very last period - Done
584,585 - consistent capitalization for woodchips and rocky

Reviewer #2: This paper is a major piece of work, extending classical descriptions of optic flow (vehicular motion) to its actual description during natural locomotion.

In order to make the contrbution "sharper", some concerns must be taken into account

1. As concerns the general paper's organization, discussion is rather large, compared to the paper itself. Parts of it might be inserted in introduction? For example, definitions of curl and divergence, that are central to the paper, were introduced by Koenderink. In the paper curl is introduced without being defined.

See next reply…

2. This is not an experimental paper per se (no real independent variable per se, besides maybe the terrain). This looks more as a high technicity instrumented observation and should be presented as such.

We have altered the wording throughout the paper to better clarify the nature of the present work, and make it clear that this is not a hypothesis driven experimental report. We hope that this alteration, along with the substantial other revisions to this manuscript will satisfy both of these points.

In addition, we have added a new appendix (Appendix B) to provide readers with an computational and intuitive introduction to the concepts of Curl and Divergence, in order to provide non-experts with sufficient footing to interpret the results of this investigation.

3. The authors started the discussion with "we have demonstrated....". This is not a demonstration but a description that leads to the suggestion that retinal flow and particularly curl motion play a critical role in heading control. This aspect of the paper is ground breaking. However, controlled experiments are needed to demonstrate this suggestion.

The text has been modified accordingly.

4. On a more functional level, the point is repeatedly made that retinal flow is the key input. What about head centered flow. In particular, is OKN totally irrelevant?

See detailed response below to Reviewer 3

5. This point is somehow mentioned in conclusion, but how might some rythmical aspects of the visual consequences of walking be present (and used) in the flow (including the retinal one)?

It seems likely that the rhythmic and predictable nature of the retinal stimulation resulting from gait modulation might facilitate learning of the self-generated flow patterns. However, we feel this needs to be explored in future work, so simply suggest that humans could learn these patterns, as seems plausible given the recent advances in Deep Learning.

6. page 5. "modal velocity across conditions of about 255 deg". Saccadic velocity? Just head velocity?

This refers to the velocity of the FoE in head-centered coordinates (as in Figure 3). We have altered the text to clarify this.

7. What is the necessity of the description of head velocity (figure 3)?

Head velocity plots help explain why the velocity of the head centered FOE is so high. See response above to Reviewer 1's second point.

8. Page 11. The max retinal divergence is linked to the body's world centered velocity. But do we really use absolute values? Don't we real of relative measures (ttc etc.)?

None of our analysis of Divergence and Curl utilizes absolute values – For divergence we refer to the maximum point of divergence in the retinal flow field at each moment in time (which corresponds to the direction of the eye's velocity vector on the ground plane), and for Curl we refer mostly to the sign of curl at the fovea (which specifies whether the walker will pass to the left or right of the point of fixation). We clarify this in the manuscript, and have added a new figure (Fig 7) that further emphasizes this point.

Minor point:
A few typos across the manuscript

Reviewer #3: Matthis and colleagues have recorded (head-centered) visual optic flow during real self-motion. The concurrent measurement of eye movements allowed them to construct (offline) an eye-centered representation of optic flow during real self-motion. The results are (at least from my point of view) unexpected and really exciting. The authors argue that not the head-centered flow is the most appropriate for navigational purposes, but rather the eye-centered version of it. Nevertheless, in their conclusion, the authors question the role of optic flow for the sensory guidance of everyday locomotion.
This study has been a tour-de-force. Experimentally, the authors recorded eye, head- and body movements during self-motion in the real world. Given the different recording devices, synchronization of time series data is a first major challenge. After having solved that, the authors constructed a series of retinal images (videos) based on sequences of head-centered optic flow, the eye movement recordings and a spherical pinhole model of the eye. From my point of view, it definitely was worth the effort. Results are surprising and definitely have the potential to trigger a full new series of experimental and theoretical studies on visual self-motion processing. Having said this, I also must say that I see a number of points that need to be addressed.

Major
First and foremost, the authors have constructed the eye centered images based on the assumption that the gain of tracking movements of an earth fixed target is 1.0. Yet, as shown by Lappe and colleagues (J Neurophysiol, 1998), this gain typically is more in the order of 0.5. This difference has the potential to change the results a lot. I assume that eye centered optic flow fields are no longer as stable over time as suggested by the authors. This is critical and needs to be discussed.

We agree that this is an important issue and have discussed it more extensively in the revised ms. However, we did not simply assume that the gain of the mechanisms stabilizing gaze is one. We calculated the image motion during fixations from the video images and observed that stabilization was very good (ie, a gain close to 1.0), as explained in Footnote 2 in the original ms and now treated in detail in Appendix A of the revised ms (see response above to Reviewer 1). For simplicity, we did not include this slippage in the calculations. We also present the data on stabilization accuracy, and mention it as a limitation on our simulations – see first paragraph in Section 2.1.3. It should be noted that the work by Lappe and colleagues that shows

low gain was observed in stationary animals and humans. When the observer is stationary the vestibular and spinal mechanisms associated with locomotion are not active (see response below), and stabilization must rely on visual mechanisms such as the OKN, ocular following, or pursuit which are driven by visual motion signals. With passive mechanisms such as OKN the gain is lower. With active pursuit Lappe and colleagues observed high gains in humans (Niemann et al, Vision Res 1999).

Second and related: the authors have a long-standing experience in measuring eye movements in the real world. Hence, they know that humans make 2-3 eye movements per second. The rather stable eye centered optic flow - as documented here - is found during smooth eye movements, but not during saccades. This, again, is critical, especially for the discussion of the results. The authors must discuss the consequences of saccades on the structure of retinal flow fields and their implication for the use of optic flow for navigation.

Saccadic eye movements do indeed add to the retinal image motion and a full understanding of the underlying neural mechanisms need to account for this. As shown by the videos and the eye movement records in Figure 1, subjects make saccades separated by periods where the eye is approximately stationary at a point in the world. The motion generated by saccades is not typically perceptually visible as a result of the mechanisms of saccadic suppression and visual stability, as is well known. The retinal image motion will create a blurred image and render the information difficult to use for either navigation or guiding footholds, and we have shown in other work that the location of gaze is tightly linked to the terrain complexity, and saccade timing linked to phase of the gait cycle. Therefore, we chose to examine the periods of stable gaze, where there is consensus that humans are collecting image information necessary for controlling locomotion. The retinal image motion caused by saccades is of course of interest, and must be dealt with by the visual system in ways that are not well understood (see McFarland et al, Nature Communications, 2015; Roth et al, Nature Neuroscience, 2015). We have now calculated the additional image motion on the ground plane engendered by a saccade and this is included in Appendix A.

Third: tracking of an earth fixed target during self-motion is not a “fixation”, but rather a smooth tracking movement. Hence, it could be called a smooth pursuit. This term, fixation, is used over and over in the manuscript. Yet, it is misleading. From my point of view, this does not call the overall results into question. But it has the potential to bring the discussion about the neural basis into a new direction. There are other brain regions involved in the control of smooth pursuit than of fixation. Hence, I recommend to reconsider the wording and adjust the discussion.

It is true that there is some ambiguity about the use of the term fixation and we have now clarified our usage in the ms. Typically, “fixation” is used to refer to the periods in between saccades when the eye is approximately stationary. In much of the eye movement research, the head is held fixed, so the eye is stable in the orbit as well as stable in space during a fixation. However, since the head is almost always moving to a greater or lesser extent in natural vision, stabilization mechanisms must operate continuously during the intervals between saccades to maintain a fixed gaze location in the world, so we have chosen to maintain the fixation terminology but to be clearer in the ms about what we meant. This is consistent with the recommendation of Lappi, Neuroscience & Biobehavioral Reviews (2016). When possible, we use “stable gaze” to avoid confusion.

During locomotion, to maintain gaze at a stable location in the world, a variety of mechanisms may be involved. Given the walker’s pattern of acceleration and deceleration, the vestibular-ocular reflex will be a big component of the stabilization. There is a question about the constant velocity component of the forward motion, but it is now thought that the vestibulo-ocular reflex is modulated by spinal mechanisms that increase the gain and improve stabilization during active locomotion, using corollary discharge signals in an anticipatory manner (eg Dietrich & Wuehr, J Neurol, 2019; Haggerty & King, Frontiers in Sys Neuroscience, 2018). Since the latency of the VOR is of the order of 10 msec, this is most likely the primary mechanism since the periods of stable gaze are only 100-300 msec and both pursuit and OKN have longer latencies. It is indeed possible that the pursuit system and the OKN systems also operate to improve stabilization. These systems are used to stabilize against image motion typically in a stationary observer, as is the case in many paradigms used in optic flow experiments. Pursuit and OKN have longer latency (around 100 msec), and are driven by retinal motion signals. However, in the case of locomotion, pursuit may be involved if it is activated in a predictive manner. All these mechanisms (vestibular, locomotor, and pursuit) must be coordinated in situations such as ball sports where people track moving objects while moving themselves so the issue is quite complex and not completely understood. We now make it clear that we are agnostic about the underlying mechanisms, since it is not directly relevant to our calculations.

The extent to which the gaze is stable is of course important, and we have calculated the magnitude of the retinal motion that is not accounted for in the simulations, as described above in response to reviewer 1.

To be honest, I am not really happy with the discussion of the recent paper by Heeger’s group (PNAS, 2020). To my understanding, the authors of the current study argue that their div-operation, i.e. the spatial derivative, is somewhat similar to the temporal derivative (i.e. acceleration rather than speed) as found in Burlingham and Heeger, 2020. Physically (and mathematically), this is not the case.

We do not suggest that the spatial derivative of flow underlying divergence and curl is 'somewhat similar' to the temporal derivative used in Burlingham and Heeger 2020. We simply point out that it is interesting that in both cases, the ambiguity associated with determining heading from retinal flow is solved by considering the higher-order structure of an instantaneous flow field. In our case, we utilize properties of the *spatial* derivative (flow div and curl), whereas Burlingham and Heeger utilize the *temporal* derivative (i.e. flow acceleration). Clearly these are very different concepts, but both *do* involve taking the derivative of the instantaneous retinal flow field – one with respect to space (x,y) and one with respect to time (t). Considering the large conceptual overlap between the Burlingham and Heeger paper and the present work, we believe this is a point worth making. We have added some language and altered some wording in the manuscript to clarify our point.

The discussion of potential neural correlates of the properties as found here could be improved. As an example, the authors argue that – based on their results and results from Strong et al., 2017 – area MST is not critical for the perception of self-motion. This would also be in line with results e.g. from Wall and Smith (2008), who showed with fMRI in humans that hVIP but not hMST responds solely to visual optic flow which is compatible with self-motion. On the other hand, the group of Angelaki and DeAngelis argue that monkey area MST (and PIVC and VPS) but not area VIP is behaviorally relevant for self-motion perception (2016). I suggest that the authors elaborate a bit on this controversy. Not lengthy, but to make clear that the involvement of both areas is currently not clear and, hence, is heavily investigated. Furthermore, I suggest to consider neurophysiological studies by the groups of Lappe and Bremmer, who had tested responses in monkey areas MST and VIP in very similar contexts, i.e. forward self-motion and concurrent (real or simulated) eye movements (2010 and 2014).

We now refer to the papers by Lappe and Bremmer and colleagues. We have referred to the work of Angelaki and DeAngeles in this section (Chen2013, Sunkara2015, Sunkara2016) and added the Chen2016 citation. We have tried to make the language clearer and that we are simply saying there is no consensus. Our main point is that it might be useful to use stimuli matched to the natural patterns to resolve some of these disagreements. Since the issue is complex and separate from the focus of the paper we felt unable to give more extensive discussion.

There were quite a few typos and also references to supplementary material that does not exist. Examples are the legend of Figure 3 (page 7/31) and line 168. I recommend careful proof-reading of the revised version.
The manuscript could be shortened a bit. An example is the introduction (elaboration of the phylogenetically old VOR). In the same vein, the last, half-page paragraph of the introduction could be more or less skipped or shifted towards the discussion. There were in-depth discussions in the Results section, which – to my taste – should be moved into the discussion. Examples are:
- Section 2.2.2, around line 243
- Section 2.2.3, lines 271 – 285
- Section 2.2.3, lines 292 – 316

We have chosen not to act on these recommendations at this point, partly because it was not mentioned by the other reviewers, and partly for reasons described in the following.
The paper has an atypical format because of the combination of novel data collection followed by simulations. In addition, it is quite complex and we have found that there are many opportunities for confusion. We were concerned in the introduction to paint the picture of what actually happens in natural navigation to underscore the differences with standard paradigms where the subject does not move. (This is also why we need the movies.) In addition, we were concerned to motivate the paper and to show how what we did differed from results in the literature, as this has also been a source of confusion in the past. The discussion in section 2.2 helps to explain the immediate implications of the calculations, and we think this helps with the exposition and the narrative. The Discussion Section takes a step back from this and is a bit more general. We think these are choices where there may not be a lot of agreement between readers, so have kept the structure as it was.

Have all data underlying the figures and results presented in the manuscript been provided?
Large-scale datasets should be made available via a public repository as described in the *PLOS Computational Biology* data availability policy, and numerical data that underlies graphs or summary statistics should be provided in spreadsheet form as supporting information.

Reviewer #1: Yes

Reviewer #2: None

Reviewer #3: None

PLOS authors have the option to publish the peer review history of their article (what does this mean?). If published, this will include your full peer review and any attached files.

If you choose "no", your identity will remain anonymous but your review may still be made public.

Do you want your identity to be public for this peer review? For information about this choice, including consent withdrawal, please see our Privacy Policy.

Reviewer #1: **Yes:** Bruno A. Olshausen

Reviewer #2: **Yes:** Daniel R Mestre

Reviewer #3: No

Figure Files:

While revising your submission, please upload your figure files to the Preflight Analysis and Conversion Engine (PACE) digital diagnostic tool, https://pacev2.apexcovantage.com. PACE helps ensure that figures meet PLOS requirements. To use PACE, you must first register as a user. Then, login and navigate to the UPLOAD tab, where you will find detailed instructions on how to use the tool. If you encounter any issues or have any questions when using PACE, please email us at figures@plos.org.

Data Requirements:

Please note that, as a condition of publication, PLOS' data policy requires that you make available all data used to draw the conclusions outlined in your manuscript. Data must be deposited in an appropriate repository, included within the body of the manuscript, or uploaded as supporting information. This includes all numerical values that were used to generate graphs, histograms etc.. For an example in PLOS Biology see here: http://www.plosbiology.org/article/info%3Adoi%2F10.1371%2Fjournal.pbio.1001908#s5.

Reproducibility:

To enhance the reproducibility of your results, we recommend that you deposit your laboratory protocols in protocols.io, where a protocol can be assigned its own identifier (DOI) such that it can be cited independently in the future. Additionally, PLOS ONE offers an option to publish peer-reviewed clinical study protocols. Read more information on sharing protocols at https://plos.org/protocols?utm_medium=editorial-email&utm_source=authorletters&utm_campaign=protocols
